# *Hemitheaaestivaria* (Hübner) (Lepidoptera: Geometridae), a Palaearctic moth, new to eastern North America

**DOI:** 10.3897/BDJ.9.e64985

**Published:** 2021-05-21

**Authors:** Christian Schmidt, Alexandre Anctil

**Affiliations:** 1 Agriculture and Agri-Food Canada, Ottawa, Canada Agriculture and Agri-Food Canada Ottawa Canada; 2 Centre de données sur le patrimoine naturel du Québec - ministère des Forêts, de la Faune et des Parcs, gouvernement du Québec, Quebec, Canada Centre de données sur le patrimoine naturel du Québec - ministère des Forêts, de la Faune et des Parcs, gouvernement du Québec Quebec Canada

**Keywords:** alien species, invasive species, exotic species, Geometrinae, citizen science

## Abstract

The geometrid moth *Hemitheaaestivaria* (Hübner, 1789) was introduced from Europe to North America, first being detected in British Columbia in 1973. Until 2019, its North American range was limited to a restricted area of the Pacific Northwest. Here, we report on the first records of *H.aestivaria* for eastern North America from three widely separated urban centres in eastern Canada during 2019-2020.

## Introduction

**Figure 1.**
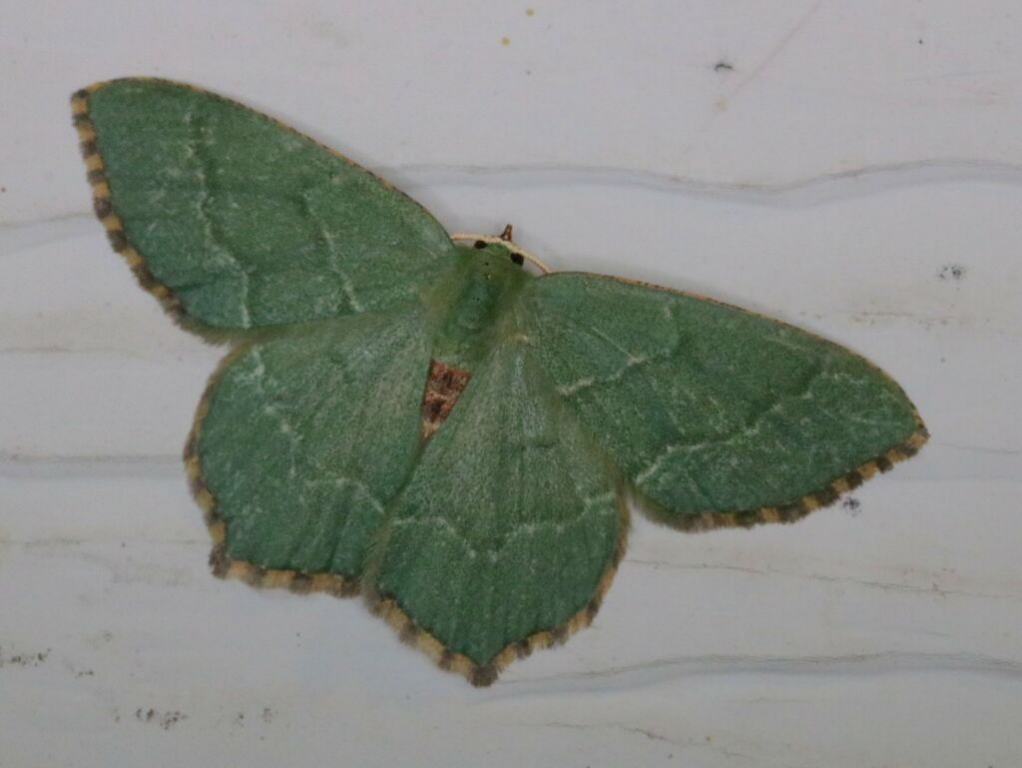
*Hemitheaaestivaria* photographed in Saint-Augustin-de-Desmaures, Québec, on 30 June 2020. Photo credit: Alexandre Anctil.

*Hemitheaaestivaria* (Hübner, 1789) (Fig. 1) is one of numerous European Lepidoptera species accidentally introduced to North America and, more specifically, one of six geometrids accidentally introduced to the Pacific Northwest Region (Pohl et al. 2016, [Bibr B6747539][Bibr B6747564]). Three of these species have had separate introductions (of independent European origin or from within North America): *Operophterabrumata* (L.), *Pasiphilarectangulata* (L.) and *Therajuniperata* (L.) (Ferguson 1975, Ferguson 1978, Maier 2005). Here we report the first eastern North American records of *Hemitheaaestivaria* (Table 1), which was previously restricted to coastal British Columbia, Canada and adjacent USA.

In Europe, larvae of *Hemitheaaestivaria* feed on a broad diet of deciduous broad-leaved shrubs comprising at least seven different plant families (summarised by Hausmann 2001). In British Columbia, larvae have been recorded from cherry, apple, wild plum and *Rubus* and *Crataegus* (Bolte and Munroe 1979). It is not clear to what extent the B.C. hostplant records represent sampling bias, versus a biological preference for spring-flowering Rosaceae shrubs. Early instar larvae of *H.aestivaria* probably feed on the foliage and flowers of a wide variety of non-coniferous shrubs. In Europe and British Columbia, *H.aestivaria* is univoltine and winter diapause is in the early larval instars; it is bivoltine in Japan, exceptionally so in Europe (Hausmann 2001). The flight period in British Columbia is late June to early August. Habitats, rich in deciduous shrubs, are favoured, including mesic forest edges, gardens and parks (Hausmann 2001). No population outbreaks or damage due to defoliation by *H.aestivaria* has been reported from the Pacific Northwest, nor from Europe.

**Figure 2.**
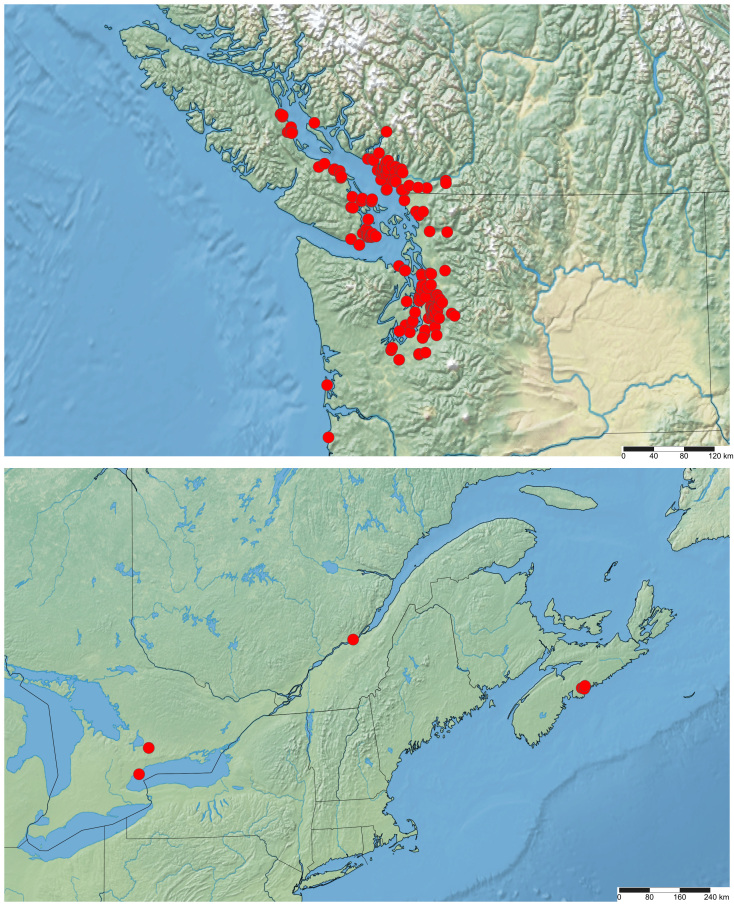
Distribution of *Hemitheaaestivaria* in western (top) and eastern (bottom) North America, based on iNaturalist (www.inaturalist.org) photographic records. Identification of all records were independently verified by the authors and are current to 11 February 2021.

The first North American specimens of *H.aestivaria* were collected in British Columbia in 1973 (Doganlar and Beirne 1979). Ferguson (1985) and Gillespie and Gillespie (1982) erroneously date the first records to 1979 and 1978, respectively (error repeated in Pohl et al. 2015, Mattson et al. 1994), but by that time, *H.aestivaria* was already common in the greater Vancouver area, with at least 40 larval collections from Burnaby, New Westminster and Langley (Doganlar and Beirne 1979). Although only documented from the Vancouver area up to 1985 by Ferguson (1985), by 1988, it had spread to eastern Vancouver Island (Taylor Bay, 17 July 1988, G.G. Anweiler, specimen # UASM59768) (University of Alberta E. H. Strickland Entomological Museum 2021) and it now occurs widely throughout the Georgia Basin (Fig. 2). The range of *H.aestivaria* also expanded southwards into the USA and it is now widespread in the Puget Sound region. On the Pacific coast, it is documented from Pacific County, WA and, in 2020, it was recorded in Oregon for the first time in Clatsop County (www.inaturalist.org/observations/53184678). However, *H.aestivaria* has not expanded into interior British Columbia or Washington east of the Cascade/Coast Range crest since establishment in the early 1970s and its North American distribution has remained restricted to a small area of the Pacific Northwest.

## First records of *Hemitheaaestivaria* for eastern North America

On 8 July 2019, an adult individual of *H.aestivaria* was photographed in the Durham Region of southern Ontario. During the summer of 2020, adults of *H.aestivaria* were photographed in Toronto, Ontario (June 29), Saint-Augustin-de-Desmaures, Québec (27 June - 24 July; up to three individuals per night) and Halifax, Nova Scotia (14 July - 26 July). These represent the first records for eastern North America (Table 1Figs 1, 2).

## Discussion

It is unclear if the eastern North American records represent new and temporary introductions or if *H.aestivaria* has been established for a number of years and simply remained undetected at low population densities. Although many iNaturalist observations of Geometridae are now available for the urban regions where *H.aestivaria* was found (> 5000 for Toronto, > 800 for Quebec City and > 800 for Halifax, as of Feb 2021), the vast majority of these are for 2018 – 2020. For example, the Moths of Ontario iNaturalist project currently has about 144,000 observations for 2020, an increase of about 70% from the previous year (https://www.inaturalist.org/projects/moths-of-ontario/stats). It is therefore possible that *H.aestivaria* has gone undetected for a number of years prior to 2020. The synchrony in appearance of such widely-disjunct localities in 2020 is noteworthy and is also perhaps best explained by a substantial increase of iNaturalist observers and observations in 2020.

The occurrence of *H.aestivaria* in the Toronto, Québec and Halifax Regions and its absence in relatively well-surveyed adjacent regions, indicates multiple points of introduction, probably via major shipping ports-of-entry along the Atlantic coast and St. Lawrence corridor or through aerial transportation. Indeed, the observations from Toronto, Saint-Augustin-de-Desmaures and Halifax are all located within 30 km of an international airport. Since both larvae and adults are relatively large and short-lived, the most plausible mode of transport is as dormant (overwintering) eggs, which could easily escape detection on fruit tree or ornamental nursery stock. DNA sequencing of eastern populations could shed light on their geogrphic origin.

To our knowledge, no study on the climatic niche of *H.aestivaria* has been conducted. In Europe, it is widespread, but absent from northern Scandinavia and the Mediterranean lowlands (Hausmann 2001). Skou (1986) reported that its distribution was limited to the southernmost coastal parts of Norway, Sweden and Finland and no significant inland range expansion has been noted since then (Global Biodiversity Information Facility 2021). Although its distribution is restricted in northern Europe, *H.aestivaria* is apparently able to tolerate relatively cold winters. Nevertheless, its European distribution, combined with the fact that it has failed to colonise inland British Columbia and Washington beyond very mild coastal regions in almost 50 years after its introduction, suggests that it is unlikely that *H.aestivaria* will expand into interior eastern North America in the near future. However, the species could become more frequent in near-shore regions along the Atlantic coast, St. Lawrence River and Great Lakes, as suggested by its Fennoscandian distribution. The coming years will provide a clearer picture of the colonisation and expansion potential of *H.aestivaria* in eastern North America, particularly with the considerable surveillance potential that citizen scientist platforms, such as iNaturalist, provide. Its spread would be facilitated through transport of dormant fruit trees or nursery stock, as is the case for another introduced geometrid (Maier 2005).

## Figures and Tables

**Table 1.** First records of *Hemitheaaestivaria* for eastern North America.

**Location**	**Year**	**Month**	**Day**	**Link to observation**
Durham, Ontario	2019	07	8	www.inaturalist.org/observations/28454987
Toronto, Ontario	2020	06	29	www.inaturalist.org/observations/51445379
Saint-Augustin-de-Desmaures, Québec	2020	06	27	www.inaturalist.org/observations/51218465
Saint-Augustin-de-Desmaures, Québec	2020	06	30	www.inaturalist.org/observations/51851347 www.inaturalist.org/observations/52109366
Saint-Augustin-de-Desmaures, Québec	2020	07	1	www.inaturalist.org/observations/51734353
Saint-Augustin-de-Desmaures, Québec	2020	07	6	www.inaturalist.org/observations/52355700
Saint-Augustin-de-Desmaures, Québec	2020	07	15	www.inaturalist.org/observations/53333395
Saint-Augustin-de-Desmaures, Québec	2020	07	24	www.inaturalist.org/observations/54346240
Halifax, Nova Scotia	2020	07	14	www.inaturalist.org/observations/53499624
Halifax, Nova Scotia	2020	07	19	www.inaturalist.org/observations/53612983
Halifax, Nova Scotia	2020	07	20	www.inaturalist.org/observations/53805400
Halifax, Nova Scotia	2020	07	26	www.inaturalist.org/observations/54574094
